# Nutrition and Mental Health: Translating Scientific Insights into Clinical Psychiatry

**DOI:** 10.1192/j.eurpsy.2026.11238

**Published:** 2026-07-31

**Authors:** M. G. Codrin, G. M. Bucea, A. V. Danciu, A. Neagu, G. M. Covaci, C. Bredicean

**Affiliations:** 1 Psychiatry Compartment, Internal Medicine Department, “Dr. Victor Popescu” Emergency Military Clinical Hospital; 2 Psychiatry Compartment, Internal Medicine Department, PhD candidate at Victor Babes University of Medicine and Pharmacy Timisoara , Timisoara, Romania; 3 Department of Neuroscience, Psychiatry Discipline, Victor Babes University of Medicine and Pharmacy Timisoara, Timisoara, Romania

## Abstract

**Introduction:**

Over the past two decades, more and more research has highlighted the pivotal role of nutrition in the development, progression, and treatment of mental health disorders. The emerging field of nutritional psychiatry suggests that dietary patterns, micronutrient intake, and gut–brain interactions significantly influence mood regulation, cognitive function, and resilience to stress. While evidence linking dietary interventions with improved outcomes in depression, anxiety and stress management is expanding, the translation of these findings into routine psychiatric practice remains limited.

**Objectives:**

The aim of this study is to analyze if there is any significant connection between our daily lifestyle, eating habits and the symptoms of anxiety and depression.

**Methods:**

The study was conducted on 157 subjects and data was collected via an online questionnaire. The questionnaire included items assessing lifestyle habits (frequency of fast-food consumption, fried foods, sugary products, alcohol intake, and physical activity) as well as self-reported symptoms related to anxiety and depression (e.g., sadness, fatigue). Responses were recorded on ordinal frequency scales. An unhealthy lifestyle score was computed for each participant by averaging the frequency of unhealthy dietary behaviors (fast food, fried foods, sugar, alcohol), with higher scores reflecting more frequent unhealthy consumption.

**Results:**

An unhealthy lifestyle score was computed based on the frequency of consuming fast food, fried foods, sugary products, and alcohol. Higher scores were significantly associated with greater fatigue (ρ = 0.27, 95% CI [0.12, 0.42], p < 0.001) and with increased sadness, though to a lesser degree (ρ = 0.18, 95% CI [0.02, 0.34], p = 0.022). In addition, physical activity showed a protective effect, being inversely correlated with sadness (ρ = –0.19, p = 0.023).

These findings highlight the role of dietary patterns and lifestyle factors in mental and physical well-being, with fatigue showing the strongest association.

**Conclusions:**

Nutritional psychiatry represents a promising adjunct to conventional psychiatric treatment. By translating scientific insights into practical strategies, psychiatrists can enrich patient care, address modifiable lifestyle factors, and contribute to a more holistic model of mental health. Future directions include the development of standardized guidelines and training modules to support integration into everyday practice.

**Disclosure of Interest:**

None Declared

